# Silylated Tag-Assisted Peptide Synthesis: Continuous One-Pot Elongation for the Production of Difficult Peptides under Environmentally Friendly Conditions

**DOI:** 10.3390/molecules26123497

**Published:** 2021-06-08

**Authors:** Shinya Yano, Toshihiro Mori, Hideki Kubota

**Affiliations:** Iwate Drug Synthesis R&D Center, Sekisui Medical Co., Ltd., Iwate 028-7305, Japan; shinya.yano@sekisui.com (S.Y.); toshihiro.mori@sekisui.com (T.M.)

**Keywords:** peptide synthesis, chemical tag, one-pot, green chemistry

## Abstract

Addition of the silylated tag (STag) enables peptides to be highly soluble in CPME, allowing them to be used at high concentrations in a coupling reaction to enhance reactivity and achieve effective synthesis of sterically hindered peptides. We described the development of a continuous one-pot STag-assisted peptide synthesis platform as a method that provides near-stoichiometric, speedy, environmentally friendly, and scalable peptide synthesis.

## 1. Introduction

Solid-phase peptide synthesis (SPPS) has been widely used since the invention of peptide elongation on resin by Merrifield in 1963 [1]. Although SPPS using Fmoc chemistry is a convenient and useful method, heterogeneity of the solid phase and low mixing efficiency are often reflected by the retarded coupling rate [2]. Notably, peptide coupling of sterically hindered amino acids has been a synthetic challenge. Another drawback of SPPS is the issue of green chemistry. Considering the step-by-step synthesis, quantitative conversion should be a requirement for each elongation step. Both the lower reactivity and the lack of a direct analytic method for ensuring quantitative conversion make SPPS an excessive process, consuming 3–4 equivalents of a Fmoc-amino acid and a reagent [3]. Furthermore, consumption of a huge amount of *N*,*N*-dimethylformamide (DMF) or a hazardous halogenated solvent such as dichloromethane or chloroform due to the frequent washing of resin has been regarded as a major problem in SPPS [4].

In contrast, the major limitation in liquid-phase peptide synthesis (LPPS) is length. Even short peptide intermediates sometimes exhibit low solubility in organic solvents, which could impede the coupling reaction. In addition, LPPS is laborious and time-consuming in cases where purification of an intermediate is required.

**Figure 1:**
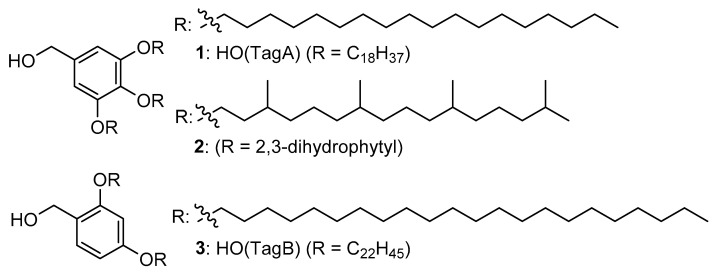
Reported chemical tags for peptides synthesis.

Recently reported chemical tags as shown in Figure 1 are expected to be part of a new tide of LPPS because peptides bearing chemical tags are prone to being soluble in an organic solvent, and near-stoichiometric coupling reactions can be realized in the resulting homogeneous solution [5]. In addition, there is the practical advantage of intermediate isolation of the chemically tagged peptides [6,7]. Addition of acetonitrile to the reaction mixture facilitates precipitation, allowing the tagged peptides to be isolated by filtration, although this protocol is not always robust when applied to a wide range of peptides. These chemical tags allowing precipitation could enhance solubility in one solvent while simultaneously diminishing solubility in another solvent. It seems contradictory that they enable both solvation and precipitation. That motivated us to develop new chemical tags, pursuing only solvation in an organic solvent. A similar approach using branched chemical tag (**2**) was recently reported, although a chloroform solvent was used in the disclosed experimental procedure [8]. Herein, we report the development of silylated chemical tags whose attachment leads to high solubilization of peptides in non-halogenated solvents and the STag-assisted peptide synthesis (STag-PS) platform which realizes high reactivity, consecutive one-pot elongation, and an environmentally friendly process.

## 2. Results

**Figure 2:**
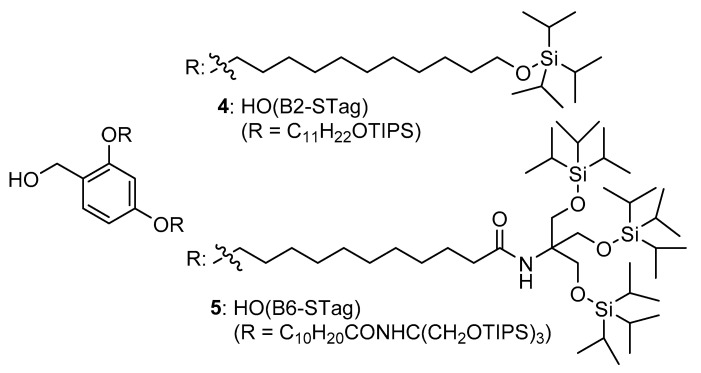
Structure of B2-STag and B6-STag.

New chemical tags were designed to have low viscosity and high hydrophobicity (Figure 2). As silyl groups are empirically known to reduce viscosity, attachment of a chemical tag bearing the triisopropylsilyl (TIPS) group is expected to provide peptides with sufficient solubility in a hydrophobic organic solvent. The terminally silylated B2-STag (**4**) was an oil, whereas tag (**3**) was reported as a solid. The solubility of peptides bearing several chemical tags was assayed by a chemiluminescent nitrogen detector (CLND) [9]. As shown in Table 1, the *N*-Fmoc-protected peptide showed lower solubility than the unprotected peptide in all the tested solvents. Compared to other chemical tags, the STagged peptide generally showed good solubility. Notably, the STagged peptides were dissolvable in cyclopentyl methyl ether (CPME) at over 100 mM in concentration and were more soluble in the mixed solvent (CPME/DMF = 7/3, *v*/*v*). The number of silyl groups affected peptide solubility, as shown by comparing the solubilities of Fmoc-FLG-O(B6-STag) and Fmoc-FLG-O(B2-STag) in CPME.

High solubility of STagged peptides could lead to their use in high concentration in coupling reactions. We then evaluated the kinetics of such a coupling reaction. A difficult coupling reaction was selected as a model reaction because the coupling reaction for standard peptides was usually completed too quickly, within 10 min, which poses challenges for analysis. As shown in Table 2, the coupling reaction between a sterically hindered H-(Aib)FL-O(B2-STag) (**6**) and Fmoc-Aib-OH (1.5 eq.) with (1-cyano-2-ethoxy-2-oxoethylidenaminooxy)dimethylaminomorpholinocarbenium hexafluorophosphate (COMU) at different concentrations was monitored by HPLC. Comparing 10 and 100 mM concentrations of STagged peptides, the higher concentration clearly resulted in a faster reaction. Interestingly, the mixed solvent (CPME/DMF) showed a slightly faster rate than DMF in this coupling reaction, although the solvent effect in amidation is not fully understood [10]. The coupling in CPME was sluggish due to the poor solubility of COMU.

**Figure 3:**
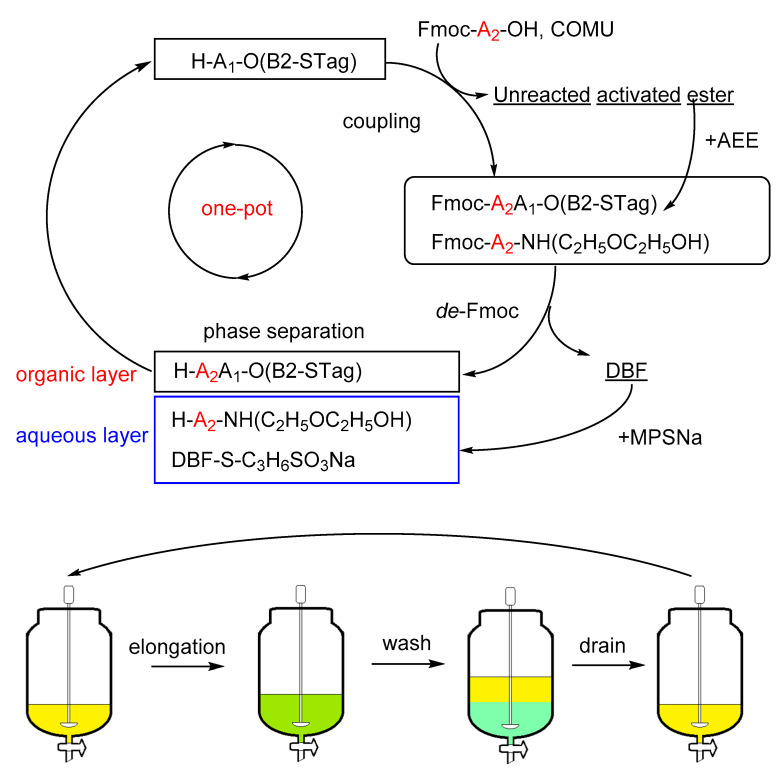
The STag-PS platform developed for continuous one-pot elongation.

Next, we envisioned a one-pot process including coupling, Fmoc cleavage, and intermediary purification. The use of phase separation instead of precipitation as an isolation step has been reported by some groups [11,12], and we adapted the protocol of Takahashi et al. to STag-PS [8]. The platform we developed is illustrated in Figure 3. Starting from the CPME solution of the STagged peptide, the elongated STagged peptide is obtained in a continuous manner after one cycle. We considered that the major risk of this one-pot process would be the generation of a double hit in the Fmoc cleavage step because of the use of 1,8-diazabicyclo(5.4.0)undec-7-ene (DBU). As shown in Table 3, the use of ethyl cyanohydroxyiminoacetate (Oxyma) resulted in the generation of a double hit. To mitigate the risk, especially when high quality is required for drug use, the residual activated carboxylic acid derivatives should be fully quenched. Immediately after the completion of the coupling reaction was ensured, the quenching by either 2-(aminoethoxy)ethanol (AEE) or 2-aminoethyl hydrogen sulfate (AEHS) succeeded in reducing the double hit. In the following Fmoc cleavage step, generated dibenzofulvene (DBF) could be trapped by sodium 3-mercaptopropylsulfonate (MPSNa) to yield a water-soluble adduct. Phase separation effectively removed all kinds of odd waste generated during the course of elongation. The ^1^H-NMR chart of the residue after the concentration of the organic layer in the elongation from H-F-O(B2-STag) (**8**) to H-FF-O(B2-STag) (**10**) proved the quantitative conversion with the removal of odd waste (see the SI for ^1^H-NMR chart). It is interesting to note that B2-STagged dipeptide (**10**) was so stable that the diketopiperazine formation was almost negligible (see the SI for ^1^H-NMR chart). Coupling reagents such as 1-ethyl-3-(3-dimethylaminopropyl)carbodiimide hydrochloride (EDCI) and 4-(4,6-dimethoxy-1,3,5-triazin-2-yl)-4-methylmorpholinium chloride (DMT-MM) are compatible with the STag-PS platform because waste can be washed out during phase separation. In addition, the cleavage of B2-STag proceeded smoothly under mild acidic conditions.

The key in the STag-PS platform in this study is the use of CPME as a solvent. CPME has unique properties as it is light and hardly miscible with water, forming azeotropes with water [13]. Clear phase separation between organic and aqueous layers allowed the product loss to be minimized (about 0.1–0.5%). Low density of CPME ensured that aqueous washes were simple to carry out and less laborious. The protocol allows the aqueous layer to be simply drawn from the bottom of the reactor to a waste tank, making STag-PS a real one-pot process (Figure 3). In addition, the intermediary STagged peptide residue could be azeotropically dried by concentrating CPME. This simple drying operation is quite useful for the difficult coupling reaction, which requires a long reaction time [14].

Macrocyclic peptides possessing as high affinity as antibodies have recently attracted interest as potential drug candidates. The constrained peptides containing many *N*-methylamino acids, such as cyclosporine, have attracted particular attention because of their high metabolic stability and membrane permeability [15,16]. This trend has illuminated a synthetic challenge for sterically hindered peptides containing successive *N*-methylamino acids because attempts to couple hydrophobic *N*-methylamino acids and bulky *N*-methylated peptides by reaction in SPPS have often given poor results [17,18]. The development of an effective coupling method has been an urgent issue for drug development.

To further demonstrate the potential of STag-PS, we applied it to the synthesis of sterically hindered peptides and evaluated the content of deletion sequences. Starting from the loading to B2-STag (**4**), Fmoc-amino acids including *N*-methylamino acid were consecutively elongated (Table 4). The coupling reaction with the fourth Fmoc-*N*-methylamino acid and the fifth Fmoc-tyrosine was carried out after a simple drying operation by using 1.8 equivalents of Fmoc-amino acid and COMU. Global deprotection with the trifluoroacetic acid (TFA) cocktail followed by the precipitation in *tert*-butyl methyl ether (TBME) and filtration resulted in a crude peptide, which was analyzed by HPLC. The overall purity of H-Y(MeL)(MeL)FL-OH was 96.6% and the total uncoupled impurities of the des-MeL was 1% [19]. The more difficult H-Y(MeF)(MeF)FL-OH was obtained at > 90% purity.

## 3. Discussion

A major limitation of SPPS arises from low solubility of peptides and the main role of attached chemical tags should be to enhance solubility in organic solvents. Whereas the first generation of chemical tags bearing a long alkyl chain has been reported to show good solubility in non-halogenated solvents, long alkane is generally viscous. Therefore, we hypothesized that low-viscous tags which possess a hydrophobic silyl group could improve solubility. Importantly, a solvent immiscible with water is preferable to develop a one-pot process; therefore, we targeted CPME as the solvent for dissolving peptides. STagged peptides showed better solubility in CPME compared to previous tags. A chemical tag bearing a branched alkyl chain was reported, but it required chloroform as a standard solvent [8]. STag, by contrast, does not require use of a halogenated solvent, thus providing an environmentally friendly elongation process. Furthermore, the one-pot process operation of the STag-PS platform is simpler and more practical than that using chloroform (Figure 3).

We speculate that high reactivity in STag-PS is probably due to the frequent collisions originating from both the liquid-phase reaction and the concentrated conditions. High concentration is beneficial not only for reactivity but for mass production because a large reactor is not required. Furthermore, the STag-PS platform is amenable to scale-up. It can be carried out similarly in either a flask or a separating funnel at laboratory scale and in a reaction tank for large production.

The developed STag-PS fits in with the green concept [20]. It can provide an alternative to SPPS, which consumes a huge amount of DMF, which is restricted by the European Regulation of Registration, Evaluation, Authorisation and Restriction of Chemicals (REACH). STag-PS uses green CPME as the main solvent and a small amount of DMF to dissolve coupling reagents. The estimated total amount of DMF used in STag-PS is about 1% of SPPS. It should be noted that a green solvent *N*-formylmorpholine reported by Albericio et al. can be used in place of DMF to realize fully the green process in STag-PS [21]. The application of long peptide synthesis will be disclosed in the future.

## 4. Materials and Methods

All the reagents were obtained from commercial sources and used without further purification. The solvents were not anhydrous-grade. Flash chromatography was performed using Kanto Chemical silica gel 60N (spherical, neutral). JEOL JNM-ECX400P (JEOL, Japan) was used to record ^1^H NMR and ^13^C NMR spectra. Chemical shifts for ^1^H NMR spectra were reported as δ in parts per million (ppm) downfield from SiMe_4_ (δ 0.0) and relative to the signal of CDCl_3_ (δ 7.26, singlet), while ^13^C NMR spectra were reported as δ in ppm downfield from SiMe_4_ (δ 0.0) and relative to the signal of CDCl_3_ (δ 77.16, triplet). HPLC and MS spectra were recorded on a Shimadzu LC20A system (Shimadzu Corporation, Japan) and LCMS-2010EV or a Shimadzu LC20A XR system and amaZon SL, using a C18 column (YMC-Pack Pro C18, 5 µm, 250 mm × 4.6 mm ID; MonoBis low-pressure type, 11 nm, 150 mm × 3.2 mm ID; Kinetex 5 µm EVO C18 100 Å LC Column, 250 mm × 4.6 mm ID; or Kinetex 1.7 µm EVO C18 100 Å LC Column, 100 mm × 2.1 mm ID) with detection at 215 nm. Chemiluminescent nitrogen detector (CLND) spectra were recorded on a Shimadzu LC10A system and Antek 8060 (PAC, Houston, TX, USA). High-resolution mass spectra (HRMS) were recorded on Bruker ultrafleXtreme (Bruker, Billerica, MA, USA). (Copies of the ^1^H-NMR and HPLC charts and experimental data for compounds.are shown in Supplementary Materials)

### 4.1. Loading: Preparation of H-F-O(B2-STag) (**8**)

To the solution of HO(B2-STag) (**4**) (10.0 g, 12.6 mmol) in CPME (177 mL), Fmoc-amino acid (14.6 g, 37.8 mmol) in DMF (76 mL), EDCI (7.25 g, 37.8 mmol), and DMAP (0.15 g, 1.26 mmol) were successively added. The reaction mixture was stirred for 30 min at room temperature. After ensuring completion of the reaction by HPLC, AEE (3.0 mL, 30.2 mmol) was added, and the reaction mixture was stirred for 15 min. A solution of MPSNa (53.9 g, 302 mmol) in dimethyl sulfoxide (DMSO) (277 mL) was added. Then, the mixture was cooled to 10 °C, DBU (23 mL, 151 mmol) was added, and the reaction mixture was stirred for 30 min. After neutralization with 4 M HCl/CPME (40 mL, 159 mmol), the organic solution was successively washed with the mixture of 20% NaClaq and 10% Na_2_CO_3_aq and with the mixture of 50% K_2_HPO_4_aq and a small amount of DMF and DMSO. The solution was concentrated and the crude residue was purified by silica gel chromatography (Hep/AcOEt = 7/1–3/1) to afford H-F-O(B2-STag) (**8**) (10.8 g, 11.5 mmol, 91%, yellow oil).

^1^H NMR (CDCl_3_) δ 0.98–1.13 (42H, m), 1.22–1.39 (24H, m), 1.39–1.49 (4H, m), 1.49–1.58 (4H, m), 1.72–1.82 (4H, m), 2.82–2.91 (1H, m), 3.04–3.12 (1H, m), 3.62–3.69 (4H, m), 3.73 (1H, dd, *J* = 7.8, 5.0 Hz), 3.95 (4H, t, *J* = 6.9 Hz), 5.14 (2H, s), 6.39–6.49 (2H, m), 7.10–7.30 (6H, m). ^13^C NMR (CDCl_3_) δ 12.0, 18.0, 25.8, 26.1, 26.1, 29.2, 29.3, 29.4, 29.4, 29.5, 29.5, 29.5, 29.6, 29.6, 29.6, 29.6, 33.0, 40.8, 55.7, 62.4, 63.5, 68.1, 99.6, 104.5, 116.2, 126.7, 128.5, 129.4, 131.5, 137.2, 158.6, 160.9, 175.2. HRMS (MALDI): calculated for C_56_H_101_NNaO_6_Si_2_ [M + Na]^+^: 962.7060, found: 962.7059.

### 4.2. STag-PS: Synthesis of H-FF-O(B2-STag) (**10**)

H-F-O(B2-STag) (**8**) (1.00 g, 1.06 mmol) was dissolved in CPME/DMF (4/1, 21.3 mL). Fmoc-F-OH (0.49 g, 1.27 mmol), COMU (0.54 g, 1.27 mmol) and DIPEA (0.74 mL, 4.24 mmol) were successively added to the solution. The reaction mixture was stirred for 30 min at room temperature. After completion of the reaction, AEE (26 μL, 0.25 mmol) was added, and the reaction mixture was stirred for 15 min at room temperature. A solution of MPSNa (0.29 g, 1.63 mmol) in DMSO (1.4 mL) and solid MPSNa (0.16 g, 0.92 mmol) were added. After cooling to 10 °C, DBU (0.83 mL, 5.53 mmol) was added, and the reaction mixture was stirred for 30 min. After neutralization with 1N H_2_SO_4_aq (6.4 mL, 6.4 mmol), the organic layer was successively washed with water and the mixture of 50% K_2_HPO_4_aq and DMF. The solution was concentrated and the residue was dried in vacuo for 24 h to afford H-FF-O(B2-STag) (**10**) (1.15 g, 1.06 mmol, 99%, yellow oil).

^1^H NMR (CDCl_3_) δ 1.00–1.13 (42H, m), 1.23–1.39 (24H, m), 1.39–1.48 (4H, m), 1.48–1.58 (4H, m), 1.73–1.83 (4H, m), 2.57 (1H, dd, *J* = 13.7, 9.6 Hz), 3.02–3.19 (3H, m), 3.58 (1H, dd, *J* = 9.6, 4.2 Hz), 3.63–3.70 (4H, m), 3.95 (1H, t, *J* = 6.4 Hz), 4.88–4.95 (4H, m), 5.15 (2H, q, *J* = 11.5 Hz), 6.40–6.47 (2H, m), 6.91–6.97 (2H, m), 7.12–7.28 (7H, m), 7.28–7.34 (2H, m), 7.72 (1H, d, *J* = 8.7 Hz). ^13^C NMR (CDCl_3_) δ 12.0, 18.0, 25.8, 26.1, 29.2, 29.3, 29.4, 29.4, 29.5, 29.5, 29.6, 29.6, 29.6, 29.6, 33.0, 38.0, 40.8, 52.6, 56.3, 62.8, 63.5, 68.1, 68.2, 99.6, 104.5, 115.9, 126.9, 128.3, 128.7, 129.4, 129.4, 131.6, 136.0, 137.8, 158.6, 160.9, 171.5, 173.7. HRMS (MALDI): calculated for C_65_H_110_N_2_NaO_7_Si_2_ [M + Na]^+^: 1109.7744, found: 1109.7776.

### 4.3. General Procedure of STag-PS

To the solution of STagged peptide* (1.26 mmol) in CPME (8.8 mL), Fmoc-amino acid (1.51 mmol) in DMF (3.8 mL), *N*,*N*-diisopropylethylamine (DIPEA) (5.04 mmol), and COMU (1.51 mmol) were successively added. The reaction mixture was stirred for 30 min at room temperature. After completion of the reaction (monitored by HPLC), AEE (0.30 mmol) was added, and the reaction mixture was stirred for 15 min. A solution of MPSNa (3.02 mmol) in DMSO (2.5 mL) was added. Then, the mixture was cooled to 5 °C, DBU (6.55 mmol) was added, and the mixture was stirred for 30 min. After neutralization with 1 N H_2_SO_4_aq (7.6 mmol), the separated organic layer was successively washed with water and the mixture of 50% K_2_HPO_4_aq and DMF to afford the elongated peptide solution. (* The loading of STag was performed similarly as shown in Table 5).

Deprotection protocol: The STagged peptide was added to 50 mM TFA cocktail (TFA/triisopropylsilane/3,6-dioxa-1,8-octanedithiol/H_2_O = 92.5/2.5/2.5/2.5, *v*/*v*/*v*/*v*). The reaction mixture was stirred for 2 h at room temperature and poured into *tert*-butyl methyl ether (TBME) cooled to 5 °C. The obtained precipitate was filtered and washed with TBME. The residue was dried in vacuo to afford the deprotected peptide.

## 5. Conclusions

The addition of STag enables peptides to become highly soluble in the green solvent CPME. The coupling reaction can be carried out at high concentration in the mixed solvent, which was revealed to enhance reactivity and allowed the synthesis of sterically hindered peptides with excellent purity. In addition, intermediate purification by phase separation allowed for repetitive peptide elongation to be carried out as a continuous one-pot process which is highly practical in terms of speed and scalability. The STag-PS platform developed in this study provides a greener process in terms of waste prevention of the solvent and benign chemistry with further advantages of a near-stoichiometric reaction and a reduction in derivatives.

## Figures and Tables

**Table 1:** Solubility of tagged peptides.

	Peptide ^a^	Solubility (mM, 25 °C)			
		CPME	CPME/DMF (7/3)	DMF	THF
entry 1	Fmoc-FLG-O(TagA) ^b^	3	46	6	32
entry 2	Fmoc-FLG-O(TagB) ^c^	4	35	- ^d^	45
entry 3	Fmoc-FLG-O(B2-STag)	124	341	509	238
entry 4	Fmoc-FLG-O(B6-STag)	549	790	>1910	-
entry 5	H-FLG-O(TagA)	105	378	51	-
entry 6	H-FLG-O(TagB)	112	113	-	193
entry 7	H-FLG-O(B2-STag)	>1630	>2230	>2420	-
entry 8	H-FLG-O(B6-STag)	>1930	>2320	>2430	-

^a^ Purity of assayed peptides, except where otherwise noted, was above 95% by HPLC (see the SI); ^b^ 91% purity; ^c^ 93% purity; ^d^ no data.

**Table 2:**
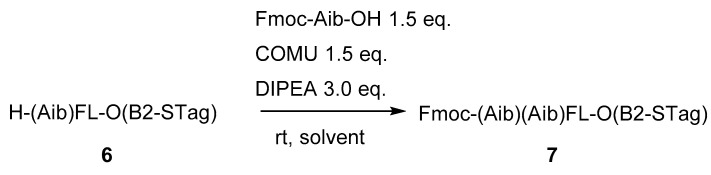
Kinetics of the coupling reaction of a STagged peptide.

	Solvent	Conc. of (6) (mM)	Conversion (%) ^a^		
			10 Min	30 Min	60 Min
entry 1	CPME/DMF (7/3)	10	43	77	91
entry 2		100	94	99	99.3
entry 3	DMF	10	27	56	74
entry 4		100	81	95	98
entry 7	CPME	10	0	1	3
entry 8		100	1	5	13

^a^ Calculated by integrating the peak area of (**6**) by HPLC.

**Table 3:**
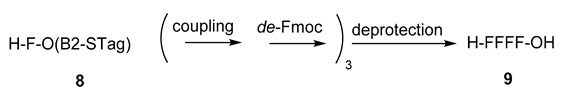
Suppression of the double hit by quenching ^a^.

	Additive; Quenching	H-FFFF-OH (%) ^b^	H-FFFFF-OH (%) ^b^
entry 1	Oxyma; none	96.8	2.8
entry 2	none; none	99.4	0.4
entry 3	Oxyma; AEE	99.6	0.3
entry 4	Oxyma; AEHS	99.7	0.1

^a^ Coupling conditions: Fmoc-F-OH (3.0 eq.), COMU (3.0 eq.), Oxyma (3.0 eq.), DIPEA (6.0 eq.), 100 mM, in CPME/DMF (7/3), rt, 45 min, quenching reagent (2.4 eq.). ^b^ Calculated by integrating the peak area by HPLC.

**Table 4:**
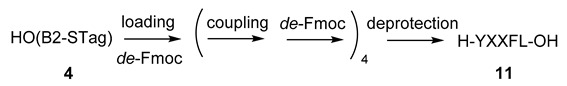
Synthesis of successive *N*-methylated peptides ^a^.

	Peptide	Purity (%) ^b^	Des-X (%) ^b^	Des-YX (%) ^b^
entry 1	H-YAAFL-OH	98.5	n.d. ^c^	n.d.
entry 2	H-Y(MeL)(MeL)FL-OH	96.6	0.4	0.6
entry 3	H-Y(MeF)(MeF)FL-OH	91.5	2.7	0.5

^a^ Coupling conditions: Fmoc-amino acid (1.2 or 1.8 eq.), COMU (1.2 or 1.8 eq.), DIPEA (4.0 eq.), 100 mM, in CPME/DMF (7/3), rt, 30 or 60 min. See the SI for details. ^b^ Calculated by integrating the peak area by HPLC. ^c^ Not detected.

**Table 5:** Conditions of general procedure and loading.

	General Procedure	Loading
substrate	STagged peptide: 1.26 mmol	STag (**4**): 1.26 mmol
Fmoc-amino acid	1.51 mmol	3.78 mmol
coupling reagent	COMU: 1.51 mmol	EDCI: 3.78 mmol
base	DIPEA: 5.04 mmol	DMAP ^a^: 0.13 mmol
AEE	0.76 mmol	3.02 mmol
MPSNa	3.02 mmol	11.34 mmol
DMSO	2.5 mL	9.5 mL
DBU	6.55 mmol	16.4 mmol

^a^ DMAP = *N*,*N*-dimethyl-4-aminopyridine.

## Data Availability

The data presented in this study are available in Supplementary Materials.

## Supplementary Materials

The following are available online. Copies of the ^1^H-NMR and HPLC charts and experimental data for compounds.

## Sample Availability

STag (**4**) are available from the authors.
