# Supplementary material for: Silylated Tag-Assisted Peptide Synthesis: Continuous One-Pot Elongation for the Production of Difficult Peptides under Environmentally Friendly Conditions
Source: Molecules. 2021 Jun 8;26(12):3497. doi: 10.3390/molecules26123497 (PMC8228865; doi:10.3390/molecules26123497)
Supplement: Supplementary file 1 [file molecules-26-03497-s001.zip › molecules-1220072-Supplementary Material.pdf]

molecules

MDPI

Supporting Information

Silylated Tag-Assisted Peptide Synthesis: Continuous One-Pot Elongation for the Production of Difficult Peptides under Environmentally Friendly Conditions

Shinya Yano, Toshihiro Mori and Hideki Kubota *

Iwate Drug Synthesis R&D center, Sekisui Medical Co., Ltd., Iwate 028-7305, Japan

Table of contents:

1. Abbreviations-- S2
2. Experimental procedure of STag-PS-- S3
   2-1. ^{1}H NMR of H-F-O(B2-STag) **8**, monitoring the reaction and ^{1}H NMR of H-FF-O(B2-STag) **10**
   2-2. Kinetics of coupling reaction
   2-3. Suppression of double hit by quenching
   2-4. Synthesis of H-YXXFL-OH
   2-5. Cleavage
   2-6. Solubility of tagged peptides
3. Sample preparation-- S8
   3-1. Preparation of B2-STag
   3-2. Preparation of B6-STag
   3-3. Preparation of Fmoc-FLG-O(B2-STag), H-FLG-O(B2-STag), Fmoc-FLG-O(B6-STag) and H-FLG-O(B6-STag)
   3-4. Preparation of Fmoc-FLG-O(TagA), H-FLG-O(TagA), Fmoc-FLG-O(TagB) and H-FLG-O(TagB)
4. References-- S13

1. Abbreviations

AcOEt = ethyl acetate
COMU = (1-cyano-2-ethoxy-2-oxoethylidenaminooxy)dimethylaminomorpholinocarbenium hexafluorophosphate
CPME = cyclopentyl methyl ether
DBU = 1,8-diazabicyclo[5.4.0]undec-7-ene
DCM = dichloromethane
DIPEA = *N*,*N*-diisopropylethylamine
DMF = *N*,*N*-dimethylformamide
DMT-MM = 4-(4,6-dimethoxy-1,3,5-triazin-2-yl)-4-methylmorpholinium chloride
Hep = heptane
IPA = 2-propanol
TagA = 3,4,5-tri(octadecanyloxy)phenylmethyl
TagB = 2,4-di(decosyloxy)phenylmethyl
MeCN = acetonitrile
MeOH = methanol
TBME = *tert*-butyl methyl ether
TFA = trifluoroacetic acid
THF = tetrahydrofuran
TIPS = triisopropropylsilyl

2. Experimental procedure

2-1. ¹H NMR of H-F-O(B2-STag) 8 and H-FF-O(B2-STag) 10

¹H NMR of H-F-O(B2-STag) **8** after purification by silica gel chromatography.

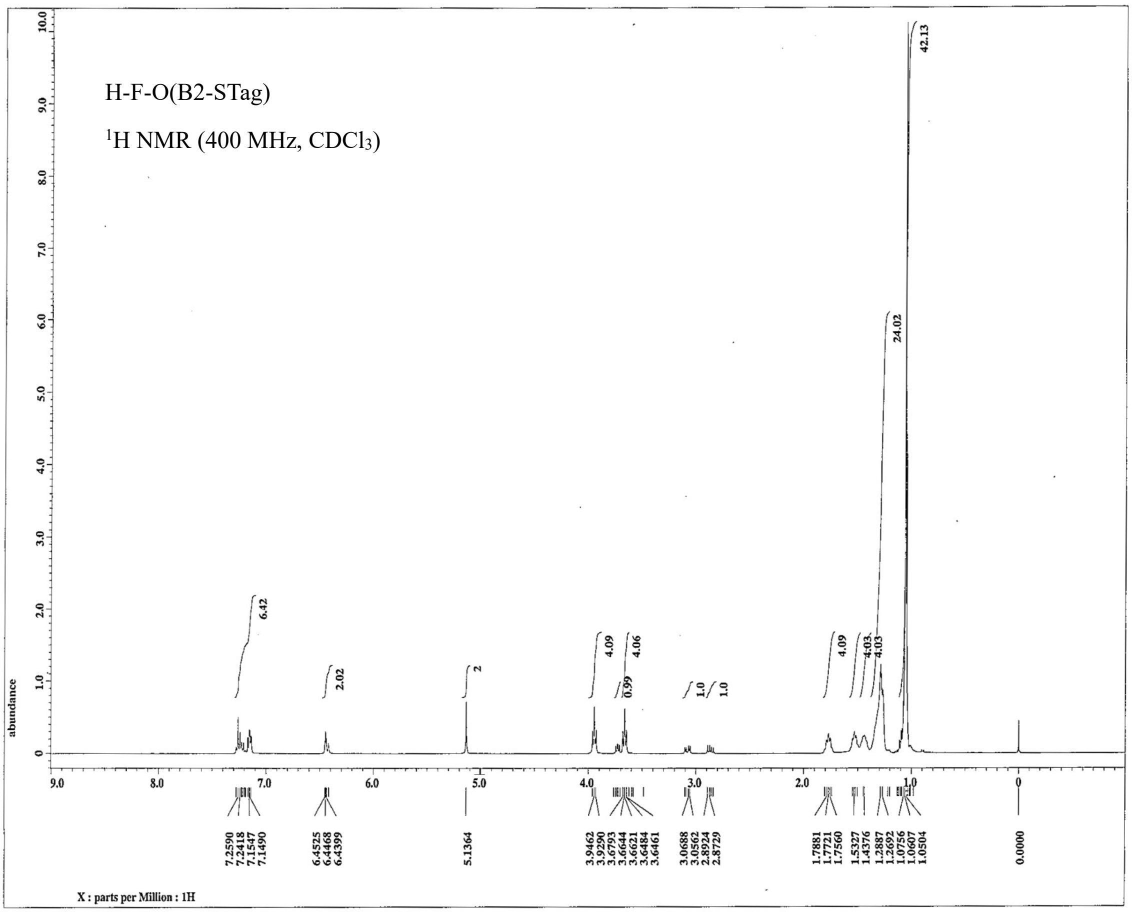

¹H NMR of H-F-O(B2-STag) **8**

Monitoring the coupling reaction and ^{1}H NMR of H-FF-O(B2-STag) **10** after phase separation

The coupling reaction was monitored by RP-HPLC (YMC-Pack Pro) with detection at 280 nm using a gradient of 75-90% B (0-10 min), 90% B (10-14 min), 90-75% B (14-15 min), 75% B (15-25 min). Eluent A: 500 mM $NaClO_4$aq; Eluent B: THF. Flow rate: 1.0 mL/min.

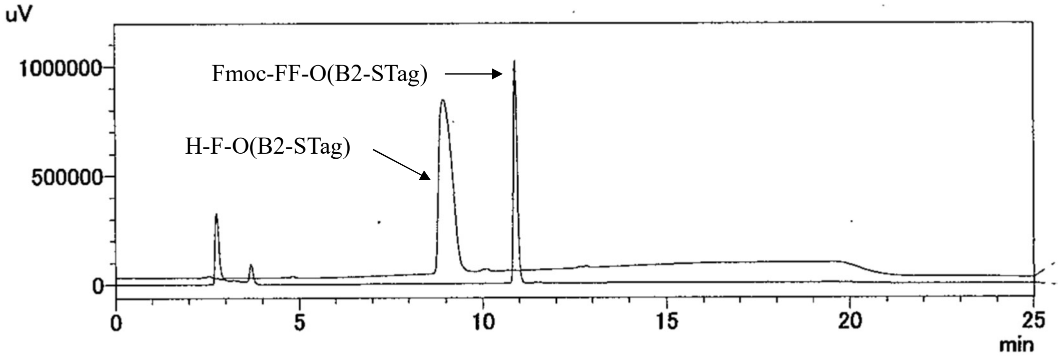

Note: *N*-unprotected peptides often give a broad peak.

HPLC chart for the monitoring the coupling reaction

^{1}H NMR of H-FF-O(B2-STag) **10** after phase separation

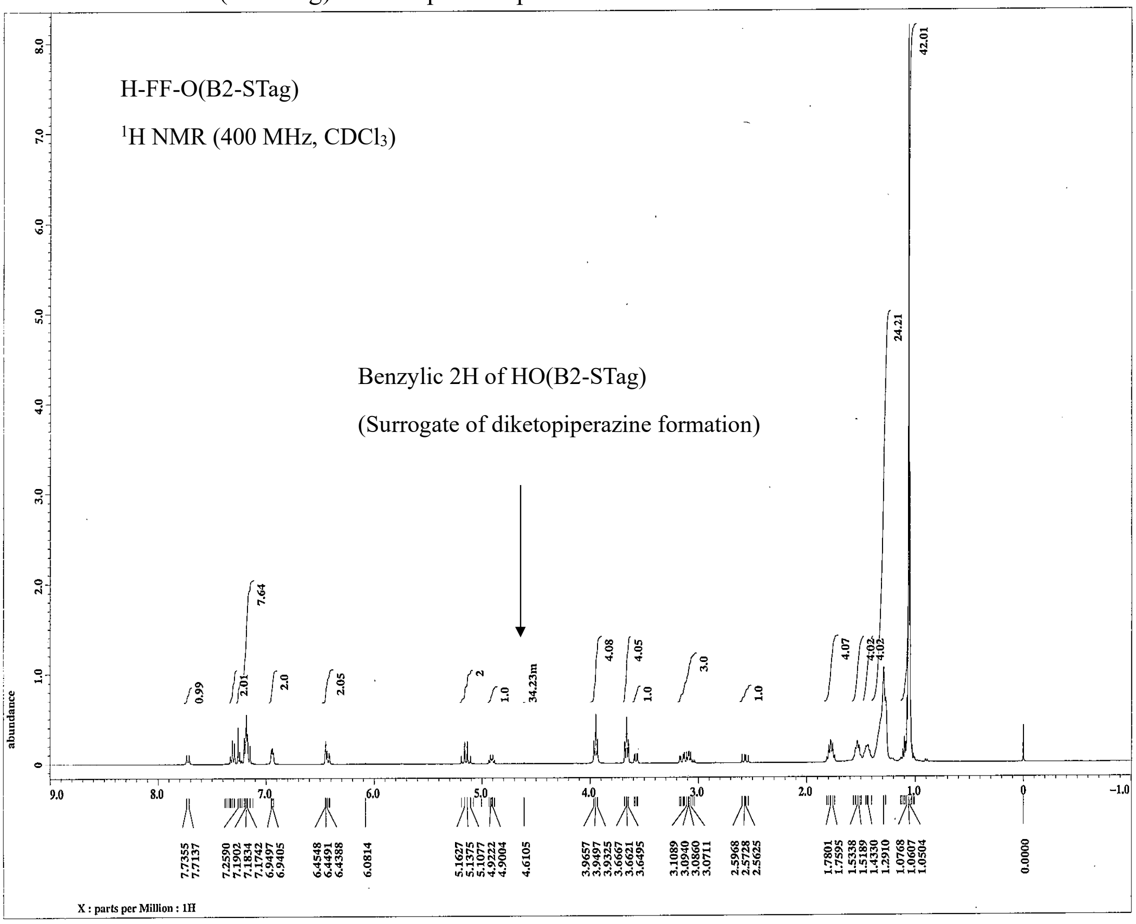

^{1}H NMR of H-FF-O(B2-STag) **10**

2-2. Kinetics of coupling reaction

Preparation of **6**.

H-O(B2-STag) ⟶ H-(Aib)FL-O(B2-STag)

4 **6**

Following the general procedure, a solution of **6** was obtained from **4** (20.0 g, 25.1 mmol). The solution was concentrated and the crude residue was purified by silica gel chromatography (Hep/AcOEt = 6/1- 1/1) to afford **6** (24.4 g, 21.4 mmol, 85%, yellow solid). ^{1}H NMR ($CDCl_3$) δ 0.86 (6H, dd, J = 6.4, 1.8 Hz), 1.00-1.12 (42H, m), 1.19 (3H, s), 1.23-1.38 (27H, m), 1.38-1.63 (11H, m), 1.71-1.81 (4H, m), 3.00-3.18 (2H, m), 3.67 (4H, t, J = 6.9 Hz), 3.89-3.96 (4H, m), 4.51-4.61 (2H, m), 5.11 (2H, q, J = 11.9 Hz), 6.41-6.45 (2H, m), 6.53 (1H, d, J = 7.8 Hz), 7.14-7.23 (4H, m), 7.23-7.30 (2H, m), 8.06 (1H, d, J = 8.2 Hz). ^{13}C NMR ($CDCl_3$) δ 12.0, 18.0, 22.0, 22.8, 24.7, 25.8, 26.0, 26.1, 28.9, 28.9, 29.2, 29.3, 29.4, 29.5, 29.5, 29.5, 29.6, 29.6, 33.0, 37.3, 41.5, 51.0, 54.1, 54.7, 62.7, 63.5, 68.1, 99.6, 104.4, 116.0, 126.8, 128.5, 129.3, 131.2, 136.9, 158.4, 160.8, 170.7, 172.5, 177.9. HRMS (MALDI): Calcd for $C_{66}H_{119}N_3NaO_8Si_2$ $[M + Na]^+$: 1160.8428, Found: 1160.8491.

H-(Aib)FL-O(B2-STag) ⟶ Fmoc-(Aib)(Aib)FL-O(B2-STag)

6 **7**

To the solution of Fmoc-Aib-OH (0.43 g, 1.32 mmol) in anhydrous DMF (26.3 mL), DIPEA (0.46 mL, 2.64 mmol) and COMU (0.56g, 1.32 mmol) were successively added at room temperature and the reaction mixture was stirred for 10 minutes. Then, the mixture was added to the solution of **6** (1.00 g, 0.88 mmol) and 1,3-dimethoxybenzene (100 mg, 0.72 mmol) as internal standard in anhydrous CPME (61.5 mL). The reaction mixture was stirred at room temperature. The conversion after 10, 30 and 60 minutes was monitored by RP-HPLC (MonoBis) using a gradient of 15% B (0-2.5 min), 15-95% B (2.5-20 min), 95% B (20-27 min), 95-15% B (27-28 min), 15% B (28-42 min). Eluent A: 5% IPAaq containing 0.1% formic acid; Eluent B: 95% IPAaq containing 0.1% formic acid. Flow rate: 0.5 mL/min.

$$\text{Conversion (\%)} = 100 - \frac{\text{(monitored HPLC Area of } \mathbf{6} \text{ / HPLC Area of 1,3-dimethoxybenzene)}}{\text{(HPLC Area of } \mathbf{6} \text{ / HPLC Area of 1,3-dimethoxybenzene without COMU)}} \times 100$$

2-3. Suppression of double hit by quenching

H-F-O(B2-STag) ⟶ H-FFFF-OH

8 **9**

Following the general procedure and deprotection protocol, the obtained crude **9** was analyzed by RP-HPLC (Kinetex 1.7 µm) using a gradient of 5% B (0-1.2 min), 5-95% B (1.2-12.8 min), 95% B (12.8-15.1 min), 95-5% B (15.1-15.2 min), 5% B (15.2-19.0 min). Eluent A: 0.1% TFAaq; Eluent B: MeCN. Flow rate: 0.3 mL/min.

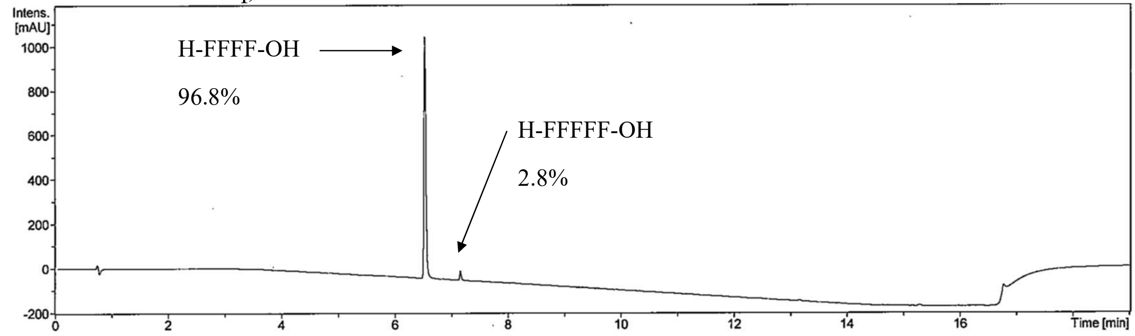

HPLC chart of H-FFFF-OH **9**

2-4. Syntheses of H-YXXFL-OH

H-O(B2-STag) ⟶ H-YXXFL-OH

4 **10**

Following the general procedure, H-XFL-O(B2-STag) solution was obtained from **4** (1.00g, 1.26 mmol). For the fourth difficult coupling reaction, the solution of STagged peptide was concentrated. The obtained residue was dissolved in CPME/DMF (anhydrous, 7:3, v/v) and then treated with Fmoc-X-OH, DIPEA (0.88 mL, 5.04 mmol) and COMU for 1 h. After *de*-Fmoc and phase separation, the obtained H-XXFL-O(B2-STag) solution was concentrated and the residue was dissolved in CPME/DMF (anhydrous, 7:3, v/v). The fifth coupling reaction was carried out with Fmoc-Y(tBu)-OH (0.87g, 1.89 mmol), DIPEA (0.88 mL, 5.04 mmol) and COMU (0.81 g, 1.89 mmol) for 1 h. After *de*-Fmoc and phase separation, the obtained H-Y(tBu)XXFL-O(B2-STag) solution was concentrated. The residue was dissolved in Hep and washed with MeCN (× 2). After concentration, the residual peptide was suffered from deprotection protocol to afford **10**.

Conditions for coupling reactions

	Fmoc-amino acid	COMU	Concentration
X = A	1.89 mmol	1.89 mmol	10 mM
X = MeL	2.27 mmol	2.27 mmol	100 mM
X = MeF	2.27 mmol	2.27 mmol	100 mM
Y(tBu)	1.89 mmol	1.89 mmol	X = A: 10 mM<br>X = MeL: 100 mM<br>X = MeF: 100 mM

H-YAAFL-OH (white solid)
RP-HPLC (Kinetex 5 µm) using a gradient of 5% B (0-5 min), 5-30% B (5-50 min), 30-95% B (50-51 min), 95% B (51-55 min), 95-5% B (55-56 min), 5% B (56-65 min). Eluent A: 0.1% TFAaq; Eluent B: MeCN. Flow rate: 1.0 mL/min.

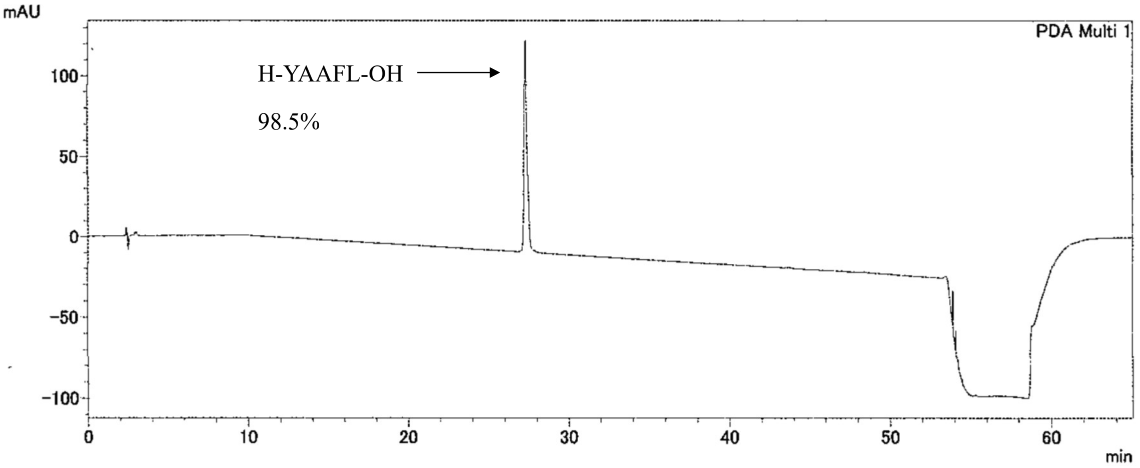

HPLC chart of H-YAAFL-OH

H-Y(MeL)(MeL)FL-OH (white solid)
HRMS (MALDI): Calcd for $C_{38}H_{57}N_5NaO_7$ $[M+Na]^+$: 718.4150, Found: 718.4169. This compound was analyzed by RP-HPLC (Kinetex 5 µm) using a gradient of 5% B (0-5 min), 5-77% B (5-32 min), 77-95% B (32-33 min), 95% B (33-37 min), 95-5% B (37-38 min), 5% B (38-47 min). Eluent A: 0.1% TFAaq; Eluent B: MeCN. Flow rate: 1.0 mL/min.

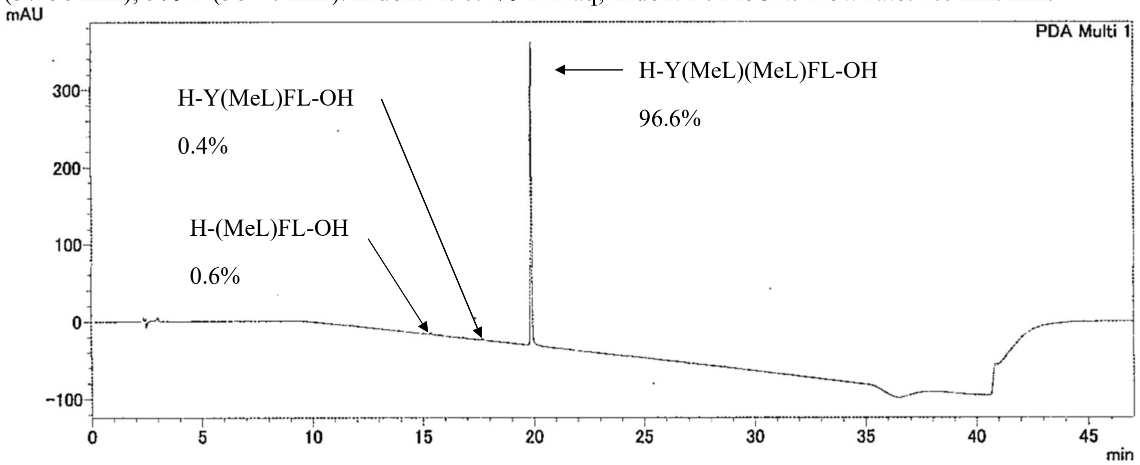

HPLC chart of H-Y(MeL)(MeL)FL-OH

H-Y(MeF)(MeF)FL-OH (white solid)
HRMS (MALDI): Calcd for $C_{44}H_{53}N_5NaO_7$ $[M+Na]^+$: 786.3837, Found: 786.3798. This compound was analyzed by RP-HPLC

(Kinetex 5 µm) using a gradient of 5% B (0-5 min), 5-77% B (5-32 min), 77-95% B (32-33 min), 95% B (33-37 min), 95-5% B (37-38 min), 5% B (38-47 min). Eluent A: 0.1% TFAaq; Eluent B: MeCN. Flow rate: 1.0 mL/min.

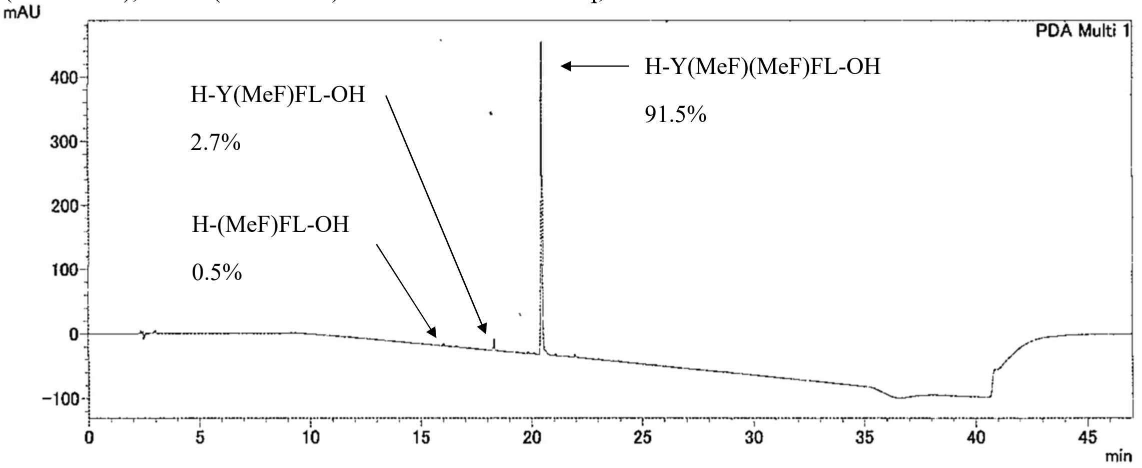

HPLC chart of H-Y(MeF)(MeF)FL-OH

2-5. Cleavage

H-FLG-O(B2-STag) ⟶ H-FLG-OH

S1 **S2**

S1 (50 mg, 45 µmol) was added to the 50mM TFA cocktail (28.3 mL, TFA/DCM/triisopropylsilane = 80/17.5/2.5, v/v/v or TFA/DCM/triisopropylsilane = 1/96.5/2.5, v/v/v). The reaction mixture was stirred at room temperature. The conversion at 0.5, 1 and 24 h was monitored by RP-HPLC (MonoBis) using a gradient of 5% B (0-5 min), 5-50% B (5-26 min), 50-95% B (26-33.5 min), 95% B (33.5-50 min), 95-5% B (50-51 min), 5% B (51-60 min). Eluent A: 0.1% TFAaq; Eluent B: MeCN. Flow rate: 0.4 mL/min.

$$\text{conversion (\%)} = \frac{(\text{HPLC Area of } \mathbf{S2} \text{ at 0.5 or 1 h})}{(\text{HPLC Area of } \mathbf{S2} \text{ at 24 h})} \times 100$$

Conversion of cleavage of B2-STag at various conditions

	**1% TFA 0.5 h**	**1% TFA 1 h**	**80% TFA 0.5 h**	**80% TFA 1 h**
H-FLG-O(B2-STag)	100 %	100 %	100 %	100 %
H-FLG-O(TagA)	0 %	0 %	66 %	100 %
H-FLG-O(TagB)	100 %	100 %	100 %	100 %

2-6. Solubility of tagged peptides

The solvent was added to the tagged peptides. The suspension was vigorously stirred for 1 h at 25 °C and centrifuged. The nitrogen content of the supernatant was measured by CLND from calibration curve of standard at 25 °C. The spectra were recorded on a SHIMADZU LC10A system and ANTEK 8060, equipped with a C18 column: widepore C18 4mm × 2.0 mm ID (Security Guard™) eluted by THF.

The assayed tagged peptides were purified beforehand. The purity of peptides is described in the following chapter.

3. Sample preparation procedure

3-1. Preparation of B2-STag

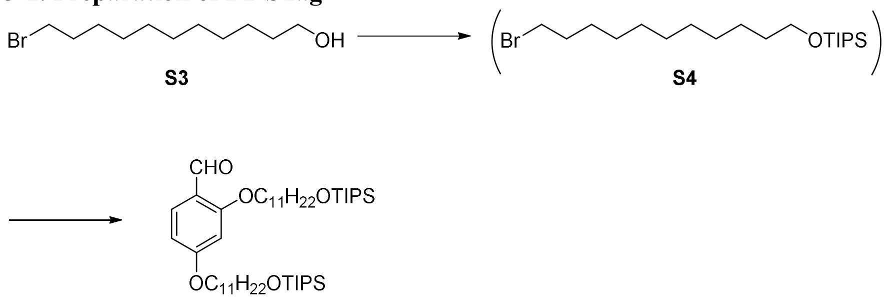

S3 S4 S5

To the solution of **S3** (0.75 g, 2.99 mmol) in DCM (11 mL), imidazole (0.51 g, 7.46 mmol) was added. The solution was cooled to 5°C and TIPSCl (0.76 mL, 3.58 mmol) was added dropwise and the reaction mixture was stirred for 2 h at room temperature. CPME was added and the solution was successively washed with 1N HClaq and water (× 4). After concentration, the residue was dissolved in Hep and washed with MeCN (× 3). The solution was concentrated and the obtained crude **S4** was suspended in DMF (8 mL). 2,4-dihydroxybenzaldehyde (0.17 g, 1.23 mmol) and K_2CO_3 (0.61 g, 4.43 mmol) were added and the reaction mixture was stirred for 2 h at 85 °C. After cooling to the room temperature, the mixture was filtrated and diluted with Hep. After phase separation, the Hep layer was successively washed with DMF (× 3), 1N HClaq, 5% $NaHCO_3$aq, water and MeCN. The solution was concentrated and the crude residue was purified by silica gel chromatography (Hep/AcOEt = 100/1-30/1) to afford **S5** (0.82 g, 1.04 mmol, 84%, colorless oil).

^{1}H NMR ($CDCl_3$) δ 0.95-1.13 (42H, m), 1.20-1.40 (24H, m), 1.40-1.57 (8H, m), 1.74-1.88 (4H, m), 3.67 (4H, t, J = 6.7 Hz), 3.97-4.06 (4H, m), 6.42 (1H, d, J = 2.3 Hz), 6.51 (1H, dd, J= 8.7, 2.3 Hz), 7.79 (1H, d, J = 8.7 Hz), 10.33 (1H, s). ^{13}C NMR ($CDCl_3$) δ 12.2, 18.2, 26.0, 26.1, 26.2, 29.2, 29.3, 29.5, 29.6, 29.7, 29.8, 33.2, 63.7, 68.6, 68.6, 99.1, 106.3, 119.1, 130.3, 163.5, 165.9, 188.6. HRMS (MALDI): Calcd for $C_{47}H_{90}NaO_5Si_2$ $[M + Na]^+$: 813.6219, Found: 813.6190.

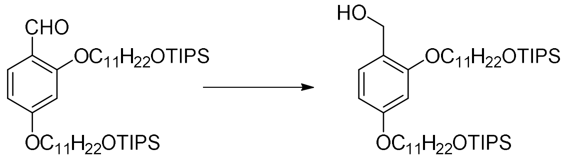

S5 4

S5 (0.49 g, 0.62 mmol) was dissolved in THF/MeOH (25/1, v/v, 5.2 mL). The solution was cooled to 5°C, $NaBH_4$ (28 mg, 0.75 mmol) was added and stirred for 1 h. CPME was added and the mixture was successively washed with 1N HClaq, 5% $NaHCO_3$aq and water. After concentration, the residue was dissolved in Hep and the Hep solution was washed with DMF (× 2) and MeCN. The solution was concentrated to afford **4** (0.44 g, 0.56 mmol, 90%, colorless oil).

^{1}H NMR ($CDCl_3$) δ 0.99-1.11 (42H, m), 1.20-1.40 (24H,m), 1.40-1.57 (8H, m), 1.70-1.85 (4H, m), 2.24 (1H, t, J = 6.9 Hz), 3.67 (4H, t, J = 6.4 Hz), 3.93 (2H, t, J = 6.4 Hz), 3.98 (2H, t, J = 6.4 Hz), 4.61 (2H, d, J = 6.4 Hz), 6.42 (1H, dd, J = 8.2, 2.3 Hz), 6.45 (1H, d, J = 2.3 Hz), 7.13 (1H, d, J = 7.8 Hz). ^{13}C NMR ($CDCl_3$) δ 12.2, 18.2, 26.0, 26.2, 26.3, 29.4, 29.4, 29.5, 29.6, 29.6, 29.7, 29.8, 33.2, 61.3, 62.2, 63.7, 68.2, 68.3, 99.9, 104.6, 121.8, 129.7, 158.3, 160.3. HRMS (MALDI): Calcd for $C_{47}H_{92}NaO_5Si_2$ $[M + Na]^+$: 815.6376, Found: 815.6373.

3-2. Preparation of B6-STag

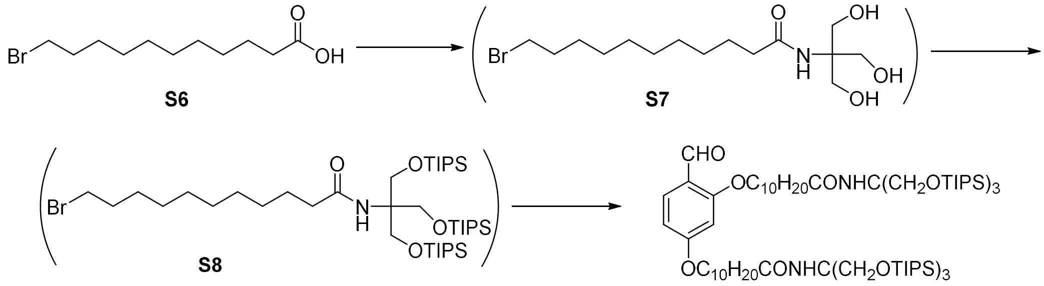

S6 S7 S8 S9

To a solution of **S6** (16.5 g, 62.2 mmol) and 2-amino-2-(hydroxymethyl)-1,3-propanediol (11.3 g, 93.3 mmol) in DMF (38 mL), DMT-MM·5H_2O (45.6 g, 124.4 mmol) and DIPEA (43.3 mL, 248.9 mmol) were added. The reaction mixture was stirred for 1 h at room temperature. AcOEt was added and the mixture was successively washed with 5% $NaHCO_3$aq, 20% NaClaq (× 3) and dried over $MgSO_4$. After concentration, the crude **S7** was dissolved in DMF (424 mL). Imidazole (26.7 g, 392 mmol) and TIPSCl (40.2

mL, 190 mmol) was added and the reaction mixture was stirred for 1 h at 85 °C. After cooling to room temperature, AcOEt was added and the mixture was successively washed with 1N HClaq, saturated $NaHCO_3$aq and 20% NaClaq, and was dried over $MgSO_4$. After concentration, the residue was pass through short silica gel chromatography (Hep/AcOEt = 25/1) to afford **S8** as colorless oil. To the suspension of the obtained **S8** in DMF (116 mL), 2,4-dihydroxybenzaldehyde (2.40 g, 17.4 mmol) and K_2CO_3 (8.65 g, 62.6 mmol) were added. The reaction mixture was stirred for 2 h at 125 °C. After cooling to room temperature, the mixture was filtered and diluted with Hep. The Hep layer was separated and successively washed with water/MeCN (1:1, v/v) and MeCN (× 5). After concentration, the residue was purified by silica gel chromatography (Hep/AcOEt = 20/1-10/1) to afford **S9** (28.4 g, 17.2 mmol, 99%, colorless oil).

^{1}H NMR ($CDCl_3$) δ 1.00-1.12 (126H, m), 1.23-1.40 (20H, m), 1.40-1.51 (4H, m), 1.51-1.62 (4H, m), 1.74-1.88 (4H, m), 2.09 (4H, t, J = 7.4 Hz), 3.97-4.07 (16H, m), 5.76 (2H, s), 6.42 (1H, d, J = 1.8 Hz), 6.51 (1H, dd, J = 8.7, 1.8 Hz), 7.80 (1H, d, J = 8.7 Hz), 10.33 (1H, s). ^{13}C NMR ($CDCl_3$) δ 11.9, 18.0, 25.8, 25.8, 26.0, 26.1, 29.1, 29.1, 29.2, 29.4, 29.5, 37.7, 61.1, 62.1, 68.4, 68.4, 98.9, 106.1, 118.9, 130.2, 163.4, 165.8, 172.5, 188.4. HRMS (MALDI): Calcd for $C_{91}H_{184}N_2NaO_{11}Si_6$ $[M + Na]^+$: 1672.2408, Found: 1672.2492.

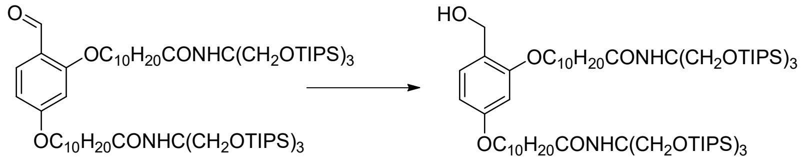

S9 **5**

S9 (28.4 g, 17.2 mmol) was dissolved in THF/MeOH (20/1, v/v, 138 mL). The solution was cooled to 5 °C, $NaBH_4$ (0.78 g, 20.6 mmol) was added and stirred for 1 h at room temperature. 1N HClaq and Hep were successively added and the mixture was successively washed with water (× 3), water/MeCN (1:1, v/v) and MeCN (× 2). After concentration, the crude residue was purified by silica gel chromatography (Hep/AcOEt = 20/1-7/1) to afford **5** (28.2 g, 17.1 mmol, 99%, colorless oil).

^{1}H NMR ($CDCl_3$) δ 1.00-1.14 (126H, m), 1.23-1.38 (20H, m), 1.38-1.50 (4H, m), 1.50-1.62 (4H, m), 1.72-1.84 (4H, m), 2.09 (4H, t, J = 7.3 Hz), 2.32 (1H, t, J = 6.4 Hz), 3.93 (2H, t, J = 6.8 Hz), 3.97 (2H, t, J = 6.4 Hz), 4.06 (12H, s), 4.61 (2H, t, J = 6.4 Hz), 5.76 (2H, s), 6.42 (1H, dd, J = 8.2, 2.3 Hz), 6.45 (1H, d, J = 2.3 Hz), 7.13 (1H, d, J = 8.2 Hz). ^{13}C NMR ($CDCl_3$) δ 11.9, 18.0, 25.8, 26.1, 26.2, 29.2, 29.3, 29.4, 29.4, 29.5, 29.6, 37.7, 61.1, 62.0, 62.1, 68.0, 68.1, 99.7, 104.4, 121.7, 129.5, 158.1, 160.1, 172.5. HRMS (MALDI): Calcd for $C_{91}H_{186}N_2NaO_{11}Si_6$ $[M + Na]^+$: 1674.2564, Found: 1674.2643.

3-3. Preparation of Fmoc-FLG-O(B2-STag), H-FLG-O(B2-STag), Fmoc-FLG-O(B6-STag) and H-FLG-O(B6-STag)

H-O(B2-STag) → Fmoc-FLG-O(B2-STag)

4 **S10**

Following the general procedure, the crude Fmoc-FLG-O(B2-STag) solution was obtained from **4** (25.1 g, 31.6 mmol). After concentration, the residue was dissolved in the mixed solution (Hep/CPME = 5.1/1, v/v). The solution was washed with MeCN (× 3) and concentrated. The residue was dissolved in THF and the solution was poured into MeCN. The generated precipitate was collected by filtration and purified by silica gel chromatography (Hep/AcOEt = 6/1-3/1) to afford **S10** (29.1 g, 21.9 mmol, 69%, yellow solid).

^{1}H NMR ($CDCl_3$) δ 0.86 (6H, dd, J = 6.4, 2.3 Hz), 1.00-1.17 (42H, m), 1.23-1.38 (24H, m), 1.38-1.49 (5H, m), 1.49-1.58 (5H, m), 1.61-1.71 (1H, m), 1.71-1.81 (4H, m), 2.99-3.15 (2H, m), 3.62-3.69 (4H, m), 3.86-4.07 (6H, m), 4.18 (1H, t, J = 6.9 Hz), 4.27-4.53 (4H, m), 5.15 (2H, s), 5.37 (1H, d, J = 6.9 Hz), 6.32 (1H, d, J = 7.3 Hz), 6.38-6.45 (2H, m), 6.49 (1H, t, J = 5.0 Hz), 7.14-7.33 (8H, m), 7.40 (2H, t, J = 7.3 Hz), 7.53 (2H, dd, J = 7.3, 5.9 Hz), 7.76 (2H, d, J = 7.8 Hz). ^{13}C NMR ($CDCl_3$) δ 12.0, 18.0, 21.8, 22.9, 24.6, 25.8, 26.0, 26.0, 29.1, 29.3, 29.3, 29.4, 29.5, 29.5, 29.5, 29.6, 29.6, 29.6, 33.0, 38.2, 40.8, 41.3, 47.0, 51.5, 56.3, 62.8, 63.5, 67.1, 68.1, 68.1, 99.7, 104.5, 115.7, 120.0, 125.0, 125.0, 127.1, 127.1, 127.8, 128.8, 129.3, 131.4, 136.1, 141.3, 143.6, 143.7, 156.1, 158.4, 161.0, 169.7, 170.9, 171.4. HRMS (MALDI): Calcd for $C_{79}H_{125}N_3NaO_{10}Si_2$ $[M + Na]^+$: 1354.8796, Found: 1354.8817. This compound was analyzed by RP-HPLC (MonoBis) using a gradient of 15% B (0-2.5 min), 15-95% B (2.5-20 min), 95% B (20-25 min), 95-15% B (25-26 min), 15% B (26-35 min). Eluent A: 5% IPAaq containing 0.1% formic acid; Eluent B: 95% IPAaq containing 0.1% formic acid. Flow rate: 0.5 mL/min.

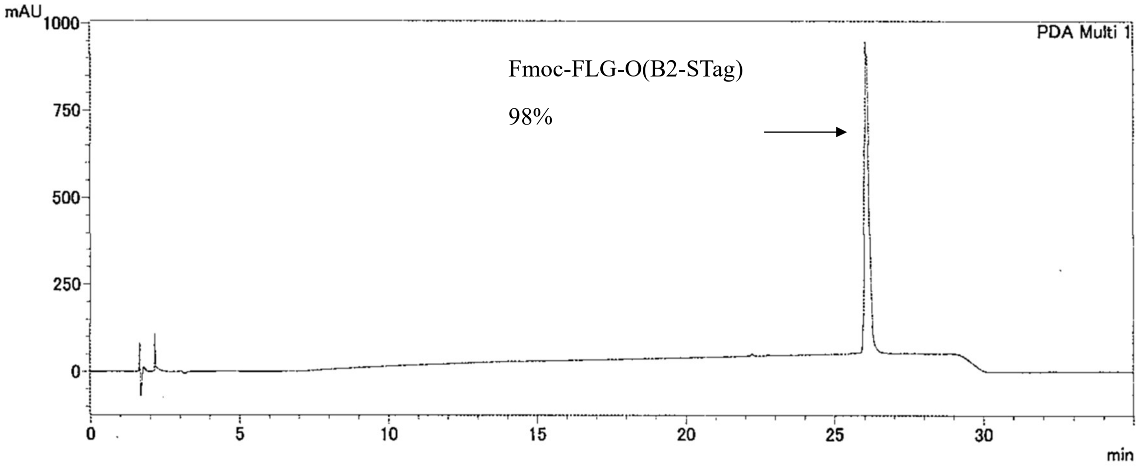

HPLC chart of Fmoc-FLG-O(B2-STag)

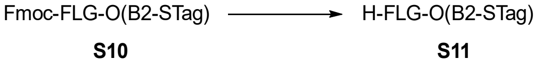

To the solution of **S10** (15.9 g, 11.9 mmol) in CPME (119 mL), DBU (3.6 mL, 23.9 mmol) was added at 5 °C and the reaction mixture was stirred for 25 minutes. After neutralization with 4M HCl/CPME (5.7 mL, 22.7 mmol), the mixture was concentrated. The residue was dissolved in Hep/CPME (2.8/1, v/v) and the solution was washed with MeCN (× 7) and concentrated. The residue was purified by silica gel chromatography (Hep/AcOEt = 5/1-1/1) to afford **S11** (10.3 g, 9.24 mmol, 78%, yellow oil).
^{1}H NMR ($CDCl_3$) δ 0.92 (6H, t, J = 6.0 Hz), 0.98-1.13 (42H, m), 1.23-1.38 (24H, m), 1.38-1.49 (4H, m), 1.49-1.62 (6H, m), 1.67-1.82 (5H, m), 2.73 (1H, dd, J = 13.7, 9.2 Hz), 3.22 (1H, dd, J = 13.3, 3.6 Hz), 3.60-3.70 (5H, m), 3.90-3.98 (4H, m), 4.01 (2H, t, J = 4.1 Hz), 4.43-4.52 (1H, m), 5.15 (2H, s), 6.39-6.46 (2H, m), 6.76 (1H, t, J = 5.5 Hz), 7.16-7.35 (6H, m), 7.67 (1H, d, J = 8.7 Hz). ^{13}C NMR ($CDCl_3$) δ 12.0, 18.0, 22.0, 23.0, 24.7, 25.8, 26.0, 26.0, 29.1, 29.3, 29.3, 29.4, 29.5, 29.5, 29.5, 29.6, 29.6, 29.6, 29.7, 33.0, 40.2, 40.6, 41.4, 51.0, 56.2, 62.9, 63.5, 68.1, 68.1, 99.7, 104.5, 115.7, 126.9, 128.7, 129.3, 131.4, 137.5, 158.5, 161.0, 169.7, 172.1, 174.8. HRMS (MALDI): Calcd for $C_{64}H_{115}N_3NaO_8Si_2$ $[M + Na]^+$: 1132.8115, Found: 1132.8173. This compound was analyzed by RP-HPLC (MonoBis) using a gradient of 15% B (0-2.5 min), 15-95% B (2.5-20 min), 95% B (20-25 min), 95-15% B (25-26 min), 15% B (26-35 min). Eluent A: 5% IPAaq containing 0.1% formic acid; Eluent B: 95% IPA containing 0.1% formic acid. Flow rate: 0.5 mL/min.

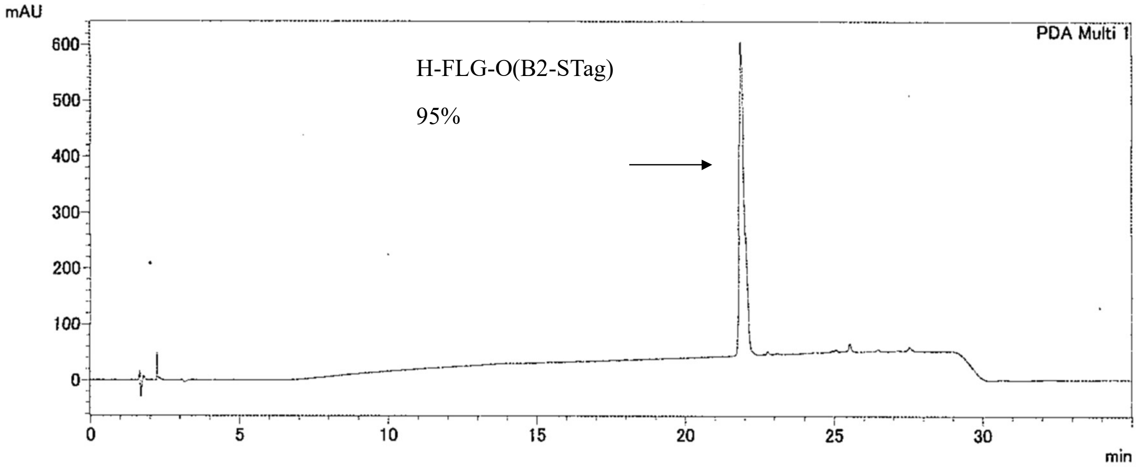

HPLC chart of H-FLG-O(B2-STag)

S12 (19.0 g, 8.67 mmol, 80% from **5**, 10.9 mmol, yellow wax); ^{1}H NMR ($CDCl_3$) δ 0.87 (6H, dd, J = 6.4, 2.3 Hz), 0.99-1.17 (126H, m), 1.22-1.37 (20H, m), 1.37-1.49 (5H, m), 1.49-1.61 (5H, m), 1.61-1.71 (1H, m), 1.71-1.80 (4H, m), 2.09 (4H, t, J = 7.4 Hz), 3.00-3.17 (2H, br), 3.82-3.97 (5H, m), 3.97-4.08 (13H, m), 4.18 (1H, t, J = 6.9 Hz), 4.26-4.52 (4H, m), 5.15 (2H, s), 5.36 (1H, d, J = 6.4 Hz), 5.76 (2H, s), 6.32 (1H, d, J = 6.9 Hz), 6.38-6.46 (2H, m), 6.49 (1H, t, J = 5.5 Hz), 7.12-7.34 (8H, m), 7.40 (2H, t, J = 7.4 Hz), 7.53 (2H, dd, J = 7.3, 5.0 Hz), 7.76 (2H, d, J = 7.3 Hz). ^{13}C NMR ($CDCl_3$) δ 11.9, 18.0, 21.8, 22.9, 24.6, 25.8, 25.8, 26.0, 26.1,

29.1,29.2, 29.3, 29.3, 29.4, 29.4, 29.5, 29.6, 29.6, 37.7, 38.1, 40.8, 41.3, 47.1, 51.6, 56.3, 61.2, 62.1, 62.8, 67.2, 68.1, 68.2, 99.7, 104.5, 115.7, 120.0, 125.0, 125.0, 127.1, 127.1, 127.8, 128.8, 129.3, 131.3, 136.1, 141.3, 143.6, 143.7, 156.1, 158.4, 161.0, 169.7, 170.8, 171.5, 172.5, 172.5. HRMS (MALDI): Calcd for $C_{123}H_{219}N_5NaO_{16}Si_6$ [M + Na]$^+$: 2213.4985, Found: 2213.5084. This compound was analyzed by RP-HPLC (YMC-Pack Pro) with detection at 280 nm, using a gradient of 75-90% B (0-10 min), 90% B (10-14 min), 90-75% B (14-15 min), 75% B (15-25 min). Eluent A: 500mM $NaClO_4$aq; Eluent B: THF. Flow rate: 1.0 mL/min.

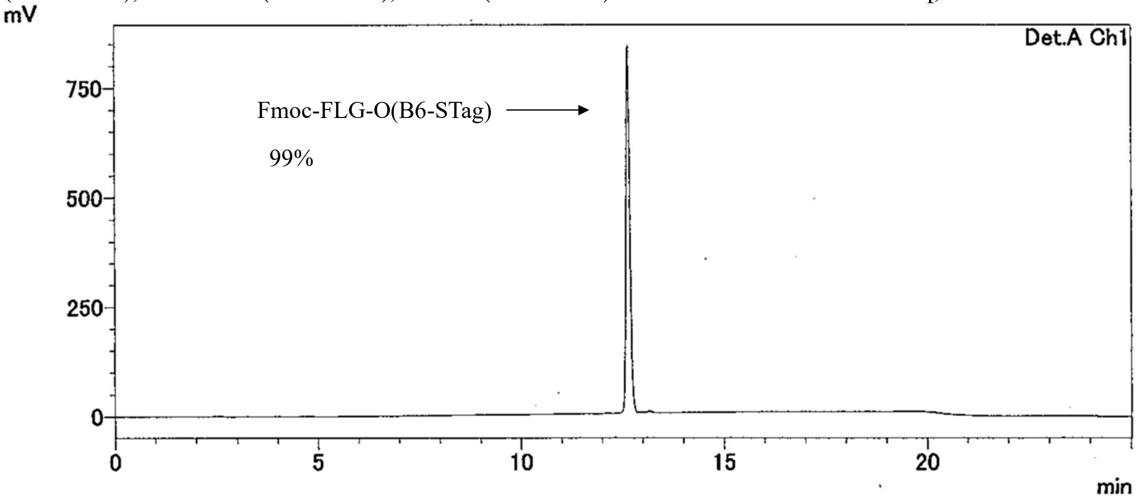

HPLC chart of Fmoc-FLG-O(B6-STag)

S13 (5.94 g, 3.01 mmol, 97% from **S12** 6.83 g, 3.11 mmol, yellow oil); ^{1}H NMR ($CDCl_3$) δ 0.92 (6H, t, J = 6.0 Hz), 0.99-1.18 (126H, m), 1.23-1.38 (20H, m), 1.38-1.49 (4H, m), 1.49-1.64 (5H, m), 1.64-1.83 (6H, m), 2.09 (4H, t, J=7.4 Hz), 2.74 (1H, dd, J= 13.3, 8.7 Hz), 3.22 (1H, dd, J= 13.7, 4.1 Hz), 3.63 (1H, dd, J= 9.2, 4.1 Hz), 3.88-3.95 (4H, m), 3.95-4.11 (14H, m), 4.42-4.52 (1H, m), 5.15 (2H, s), 5.76 (2H, s), 6.38-6.46 (2H, m), 6.75 (1H, t, J = 5.0 Hz), 7.16-7.35 (6H, m), 7.66 (1H, d, J = 8.7 Hz). ^{13}C NMR ($CDCl_3$) δ 11.9, 18.0, 22.0, 23.0, 24.7, 25.8, 25.8, 26.0, 26.1, 29.1, 29.2, 29.3, 29.4, 29.4, 29.5, 29.5, 29.6, 29.6, 37.8, 40.2, 40.6, 41.4, 51.0, 56.2, 61.2, 62.1, 62.8, 68.1, 68.2, 99.7, 104.5, 115.7, 126.9, 128.7, 129.3, 131.3, 137.5, 158.5, 161.0, 169.7, 172.1, 172.5, 172.5, 174.8. HRMS (MALDI): Calcd for $C_{108}H_{209}N_5NaO_{14}Si_6$ [M + Na]$^+$: 1991.4304, Found: 1991.4399. This compound was analyzed by RP-HPLC (YMC-Pack Pro) with detection at 280 nm, using a gradient of 75-90% B (0-10 min), 90% B (10-14 min), 90-75% B (14-15 min), 75% B (15-25 min). Eluent A: 500mM $NaClO_4$aq; Eluent B: THF. Flow rate: 1.0 mL/min.

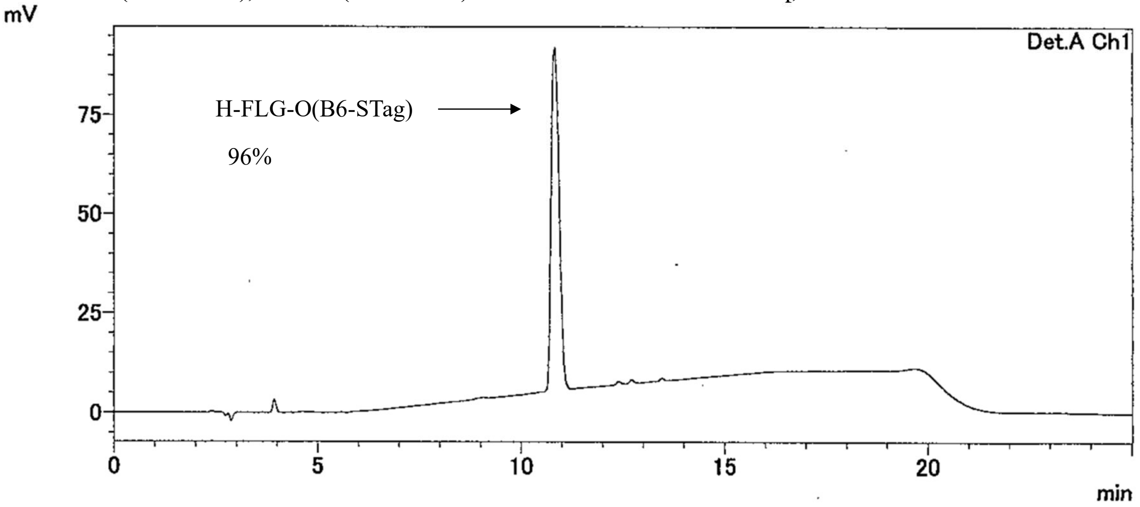

HPLC chart of H-FLG-O(B6-STag)

3-4. Preparation of Fmoc-FLG-O(TagA), H-FLG-O(TagA), Fmoc-FLG-O(TagB**), and H-FLG-O(**TagB**)**

Fmoc-FLG-O(TagA), H-FLG-O(TagA), Fmoc-FLG-O(TagB) and H-FLG-O(TagB) were obtained by the intermediate purification method of references.[1,2] Further attempt of purification by silica gel chromatography at various eluting conditions was unsuccessful, due to low solubility and hydrophobic property of the peptides.

Fmoc-FLG-O(TagA) (36.0 g, 24.8 mmol, 81% from **1**, white solid); ^{1}H NMR ($CDCl_3$) δ 0.88 (15H, t, J = 6.9 Hz), 1.22-1.38 (84H, m), 1.38-1.55 (8H, m), 1.66-1.83 (7H, m), 3.08 (2H, d, J = 5.5 Hz), 3.88-4.13 (8H, m), 4.18 (1H, t, J = 6.9 Hz), 4.30-4.52 (4H, m), 5.03 (2H, s), 5.31 (1H, d, J = 6.4 Hz), 6.30 (1H, d, J = 7.8 Hz), 6.52 (2H, s), 6.54-6.65 (1H, br), 7.14-7.35 (7H, m), 7.40 (2H, t, J = 7.3 Hz), 7.53 (2H, dd, J = 7.4, 3.2 Hz), 7.76 (2H, d, J = 7.3 Hz). ^{13}C NMR ($CDCl_3$) δ 14.1, 21.8, 22.7, 22.9, 24.6, 26.1, 29.4, 29.5, 29.7, 29.7, 30.3, 31.9, 38.0, 40.5, 41.3, 47.0, 51.5, 56.3, 67.2, 67.7, 69.1, 73.4, 107.1, 120.0, 125.0, 125.0, 127.1, 127.1, 127.2, 127.8, 128.8, 129.3, 129.9, 136.0, 138.3, 141.3, 143.6, 143.7, 153.2, 156.2, 169.6, 171.0, 171.7. HRMS (MALDI): Calcd for $C_{93}H_{149}N_3NaO_9$[M + Na]$^+$: 1475.1186, Found: 1475.1263. This compound was analyzed by RP-HPLC (MonoBis) using a gradient of 15% B (0-2.5 min), 15-95% B (2.5-20 min), 95% B (20-40 min), 95-15% B (40-41 min), 15% B (41-55 min). Eluent A: 5% IPAaq containing 0.1% formic acid; Eluent B: 95% IPAaq containing 0.1% formic acid. Flow rate: 0.5 mL/min.

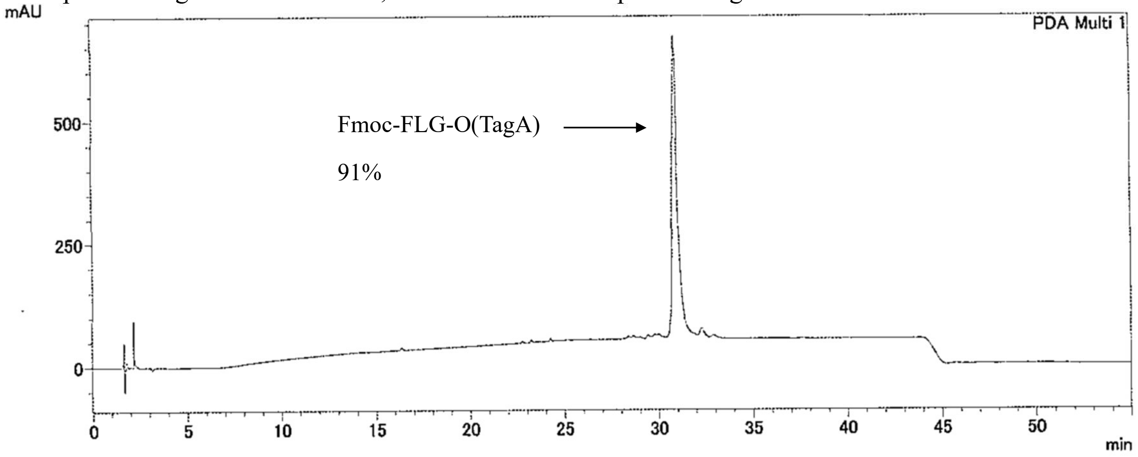

HPLC chart of Fmoc-FLG-O(TagA)

H-FLG-O(TagA) (6.23 g, 5.06 mmol, 92% from Fmoc-FLG-O(TagA) (8.00g, 5.51 mmol), white solid); ^{1}H NMR ($CDCl_3$) δ 0.84-0.96 (15H, m), 1.23-1.39 (84H, m), 1.39-1.52 (6H, m), 1.52-1.61 (2H, m), 1.61-1.83 (7H, m), 2.75 (1H, dd, J = 13.8, 9.2 Hz), 3.22 (1H, dd, J= 13.7, 4.1 Hz), 3.64 (1H, dd, J = 9.2, 4.1 Hz), 3.89-4.01 (6H, m), 4.05 (2H, dd, J= 7.8, 5.5 Hz), 4.42-4.52 (1H, m), 5.05 (2H, s), 6.52 (2H, s), 6.90 (1H, t, J = 5.0 Hz), 7.18-7.35 (5H, m), 7.68 (1H, d, J = 8.2 Hz). ^{13}C NMR ($CDCl_3$) δ 14.1, 22.0, 22.7, 22.9, 24.7, 26.1, 29.4, 29.5, 29.7, 29.7, 30.3, 31.9, 40.0, 40.5, 41.3, 51.0, 56.2, 67.7, 69.1, 73.4, 107.0, 126.9, 128.7, 129.3, 129.9, 137.4, 138.3, 153.2, 169.6, 172.2, 174.9. HRMS (MALDI): Calcd for $C_{78}H_{139}N_3NaO_7$[M + Na]$^+$: 1253.0505, Found: 1253.0554. This compound was analyzed by RP-HPLC (MonoBis) using a gradient of 15% B (0-2.5 min), 15-95% B (2.5-20 min), 95% B (20-25 min), 95-15% B (25-26 min), 15% B (26-35 min). Eluent A: 5% IPAaq containing 0.1% formic acid; Eluent B: 95% IPAaq containing 0.1% formic acid. Flow rate: 0.5 mL/min.

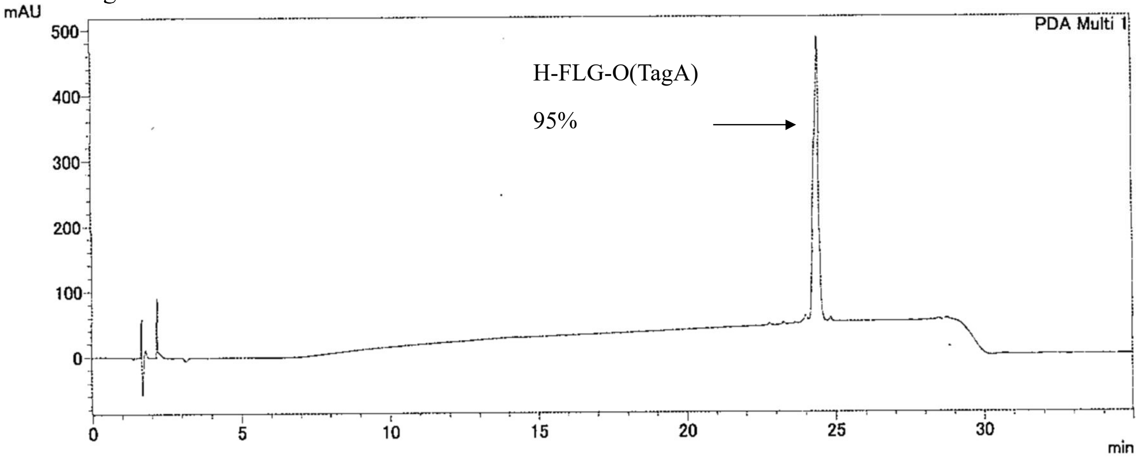

HPLC chart of H-FLG-O(TagA)

Fmoc-FLG-O(TagB) (6.50 g, 5.01 mmol, 63% from **3**, white solid); ^{1}H NMR ($CDCl_3$) δ 0.84-0.92 (12H, m), 1.20-1.37 (72H, m), 1.37-1.58 (6H, m), 1.61-1.71 (1H, m), 1.71-1.81 (4H, m), 2.99-3.15 (2H, m), 3.85-4.08 (6H, m), 4.18 (1H, t, J = 6.8 Hz), 4.26-4.53 (4H, m), 5.15 (2H, s), 5.36 (1H, d, J = 6.9 Hz), 6.34 (1H, d, J = 7.4 Hz), 6.38-6.46 (2H, m), 6.49 (1H, t, J = 4.6 Hz), 7.11-7.34 (8H, m), 7.40 (2H, t, J = 7.3 Hz), 7.53 (2H, dd, J = 7.3, 5.5 Hz), 7.76 (2H, d, J = 7.4 Hz). ^{13}C NMR ($CDCl_3$) δ 14.1, 21.8, 22.7, 22.9, 24.6,

26.0, 26.0, 29.1, 29.3, 29.3, 29.4, 29.4, 29.6, 29.7, 29.7, 31.9, 38.2, 40.7, 41.3, 47.1, 51.5, 56.3, 62.9, 67.2, 68.1, 68.2, 99.7, 104.5, 115.7, 120.0, 125.0, 125.0, 127.1, 127.1, 127.8, 128.8, 129.3, 131.4, 136.1, 141.3, 143.6, 143.7, 156.1, 158.5, 161.0, 169.7, 170.9, 171.5. HRMS (MALDI): Calcd for $C_{83}H_{129}N_3NaO_8$ [M + Na]$^+$: 1318.9672, Found: 1318.9708. This compound was analyzed by RP-HPLC (MonoBis) using a gradient of 15% B (0-2.5 min), 15-95% B (2.5-20 min), 95% B (20-40 min), 95-15% B (40-41 min), 15% B (41-55 min). Eluent A: 5% IPAaq containing 0.1% formic acid; Eluent B: 95% IPAaq containing 0.1% formic acid. Flow rate: 0.5 mL/min.

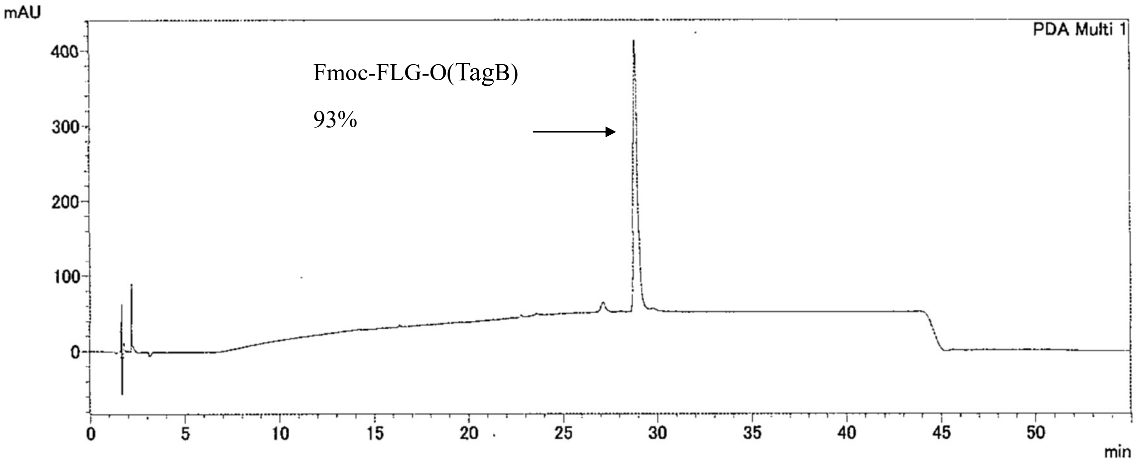

HPLC chart of Fmoc-FLG-O(TagB)

H-FLG-O(TagB) (2.44 g, 2.27 mmol, 84% from Fmoc-FLG-O(TagB) (3.52 g, 2.71 mmol), white solid); ^{1}H NMR ($CDCl_3$) δ 0.88 (6H, t, J = 6.9 Hz), 0.92 (6H, t, J = 6.0 Hz), 1.23-1.38 (72H, m), 1.38-1.49 (4H, m), 1.49-1.63 (2H, m), 1.67-1.82 (5H, m), 2.73 (1H, dd, J = 13.7, 9.2 Hz), 3.22 (1H, dd, J = 13.7, 3.6 Hz), 3.63 (1H, d, J = 6.0 Hz), 3.87-3.98 (4H, m), 4.01 (2H, t, J = 5.5 Hz), 4.42-4.52 (1H, m), 5.15 (2H, s), 6.38-6.46 (2H, m), 6.77 (1H, t, J = 5.5 Hz), 7.15-7.35 (6H, m), 7.67 (1H, d, J = 8.7 Hz). ^{13}C NMR ($CDCl_3$) δ 14.1, 22.0, 22.7, 23.0, 24.7, 26.0, 26.0, 29.1, 29.2, 29.3, 29.4, 29.4, 29.6, 29.6, 29.7, 29.7, 31.9, 40.2, 40.6, 41.4, 51.0, 56.2, 62.9, 68.1, 68.1, 99.7, 104.5, 115.7, 126.9, 128.7, 129.3, 131.4, 137.5, 158.5, 161.0, 169.7, 172.1, 174.8. HRMS (MALDI): Calcd for $C_{68}H_{119}N_3NaO_6$ [M + Na]$^+$: 1096.8991, Found: 1096.9006. This compound was analyzed by RP-HPLC (MonoBis) using a gradient of 15% B (0-2.5 min), 15-95% B (2.5-20 min), 95% B (20-25 min), 95-15% B (25-26 min), 15% B (26-35 min). Eluent A: 5% IPAaq containing 0.1% formic acid; Eluent B: 95% IPAaq containing 0.1% formic acid. Flow rate: 0.5 mL/min.

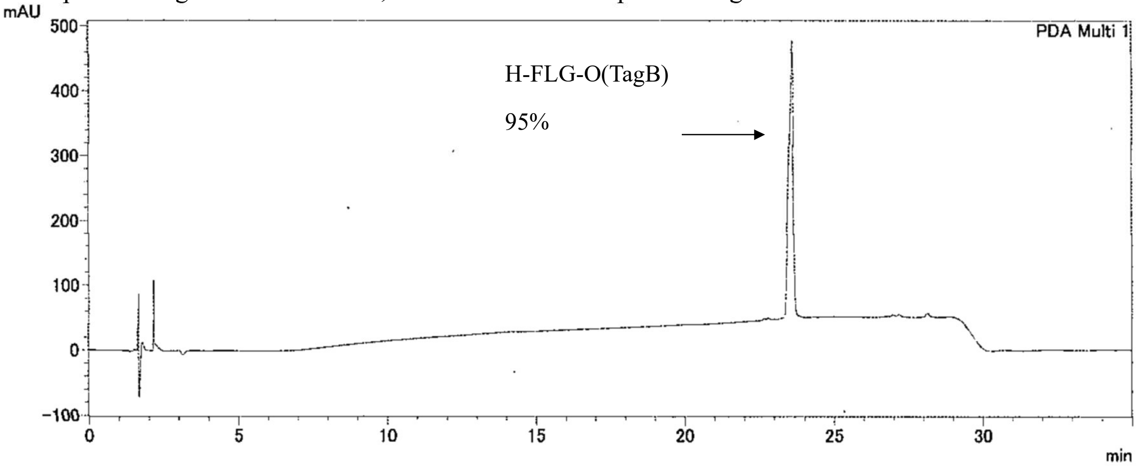

HPLC chart of H-FLG-O(TagB)

4. References

1 Tamiaki, H.; Obata, T.; Azefu, Y.; Toma, K. *Bull. Chem. Soc. Jpn.* **2001**, *74*, 733-738.
2 Okada, Y.; Suzuki, H.; Nakae, T.; Fujita, S.; Abe, H.; Nagano, K.; Yamada, T.; Ebata, N.; Kim, S.; Chiba, K. *J. Org. Chem.* **2013**, *78*, 320-327.
